# Prognostic Value of Cancer Stem Cell Marker ALDH1 Expression in Colorectal Cancer: A Systematic Review and Meta-Analysis

**DOI:** 10.1371/journal.pone.0145164

**Published:** 2015-12-18

**Authors:** Jinhuang Chen, Qinghua Xia, Bin Jiang, Weilong Chang, Wenzheng Yuan, Zhijun Ma, Zhengyi Liu, Xiaogang Shu

**Affiliations:** Department of Gastrointestinal Surgery, Union Hospital, Tongji Medical College, Huazhong University of Science and Technology, Wuhan, China; Sapporo Medical University, JAPAN

## Abstract

**Objective:**

Many studies have indicated the prognostic and clinicopathological value of aldehyde dehydrogenase 1 (ALDH1) in colorectal cancer (CRC) patients still remains controversial. Thus we performed this study to clarify the relationship between high ALDH1 expression in CRC and its impact on survival and clinicopathological features.

**Methods:**

Publications for relevant studies in Pubmed, the Cochrane Library, Embase, and China National Knowledge Infrastructure (CNKI) through April 2015 were identified. Only articles describing ALDH1 antigen with immunohistochemistry in CRC were included. The software RevMan 5.1 was used to analyze the outcomes, including 5-year overall survival (OS), disease-free survival (DFS) and clinicopathological features.

**Results:**

9 studies with 1203 patients satisfying the criteria were included. The overall rate of high ALDH1 expression was 46.5% by immunohistochemical staining. High ALDH1 expression as an independent prognostic factor was significantly associated with the 5-year OS and DFS (OR = 0.42, 95%CI: 0.26–0.68, P = 0.0004; OR = 0.38, 95%CI: 0.24–0.59, P < 0.0001, respectively). High ALDH1 expression was highly correlated with the tumor (T) stage (T3 + T4 vs. T1 + T2; OR = 2.16, 95%CI: 1.09–4.28, P = 0.03), lymph node (N) stage (N1 + N2 vs. N0; OR = 1.8; 95%CI: 1.17–2.79, P = 0.008), and tumor differentiation (G3 vs. G1 + G2; OR = 1.88; 95%CI: 1.07–3.30, P = 0.03). However, high ALDH1 expression was not significantly correlated with the patient age (>60 years old vs. <60 years old; OR = 1.11, 95%CI: 0.63–1.94, P = 0.72).

**Conclusions:**

High ALDH1 expression indicates a poor prognosis in CRC patients. Moreover, high ALDH1 expression correlates with the T stage, N stage, and tumor differentiation, but not with age.

## Introduction

Colorectal cancer (CRC) is the third commonest gastrointestinal tumors, although the diagnosis and treatments of CRC have been improving rapidly, the prognosis of patients with CRC remains poor [1]. In addition, high rates of recurrence, metastasis, and drug resistance are critical problems in CRC patients with comprehensive treatment. At present, many studies show that cancer stem cells (CSCs), a rare sub-population cancer cells, existing in various cancers such as breast cancer, lung cancer, and CRC, may be related to the above problems [2]. Several CSC markers have been identified in CRC and may cause poor outcomes of CRC [3,4]. Recently, ALDH1 is one of putative CSC marker in CRC.

The ALDH1 gene is located on chromosome 12 (12q24.2) and expresses a type of detoxifying enzyme, which contributes to the oxidation of intracellular aldehydes [5]. ALDH1 confers resistance to alkylating chemotherapeutic agents and protects against oxidative damage by catalyzing the irreversible oxidization of cellular aldehydes [6]. ALDH1 is involved in the metabolism of retinaldehyde to retinoic acid, a signaling molecule that contributes to cellular differentiation and proliferation [7]. ALDH1-positive cells with CSC properties, such as differentiation, self-renewal and tumorigenicity, have a higher capacity in xenotransplantation and chemoradiotherapy resistance and correlate with a poor prognosis of breast cancer [8]. In addition, ALDH1 activity has been shown to identify CSC-like cells in head and neck neoplasm [9]. ALDH1 appears to be a bio-marker that can be applied to isolate the CSC population in tumors obtained from patients with pancreatic cancer or CRC [7,10]. Furthermore, ALDH1 acts as a promoter, inducing epithelial-mesenchymal transition (EMT) in cancer cells [11]. EMT promotes epithelial cancer cells to obtain stemness and correlates with tumor invasion and metastasis [12]. In recent years, many studies have reported that ALDH1 expression correlates with a poor clinical prognosis in lung, prostate, pancreatic, and gastric cancers as well as in CRC [2,13]. However, among numerous independent studies, the prognostic value of ALDH1 for CRC remains controversial. Many studies have reported that ALDH1 is an independent prognostic marker associated with the clinicopathological features and poor OS in CRC [14,15]. Yet, some studies indicate that ALDH1 is not related to tumor stage or patient age [16]. Thus, this systematic review was conducted to evaluate the association between ALDH1 expression and OS, DFS as well as clinicopathological features of CRC.

## Methods

### Search strategy

All publications were identified in the following electronic databases: Cochrane Library, Pubmed, EMBase, and China CNKI up to April 2015. The search terms included “ALDH1”, or “aldehyde dehydrogenase 1” with “colon cancer,” “rectal cancer,” or “colorectal cancer”. Review articles and reference lists from the relevant articles were also screened to identify additional relevant studies. First, we excluded the unrelated studies through carefully browsing the title and abstract of each publication. Only studies that detected the expression of ALDH1 by immunohistochemical staining in CRC were included. Then, the full text of the remaining potential publications were reviewed to check whether they met the selection criteria.

### Selection criteria

The studies that evaluated the correlation between ALDH1 expression and the prognosis of CRC were included. The criteria for inclusion were as follows: 1. Diagnosis of CRC was proven by histopathological methods; 2. publications with full text defining the ALDH1-high expression by immunohistochemistry; 3. evaluation of the correlation between ALDH1 expression, 5-year OS/DFS rate, or clinicopathological features; 4. publications with adequate data to calculate the odds ratio (OR) of the effective index; 5. studies as original research articles that were published in English or Chinese. The exclusion criteria were as follows: 1. literature reviews, comments, letters, or duplicated publications; 2. no sufficient data to estimate the OR and 95%CI; or 3. the full text could not be retrieved.

To control this meta-analysis, we examined the quality of the included publications according to a rigorous evaluation system provided by the Cochrane Center. The quality evaluation criteria were as follows: 1. the study population and country were clearly defined; 2. the study design and outcome evaluation were clearly defined; 3. the evaluation method and cutoff for ALDH1 expression were clearly defined; and 4. an adequate follow-up time was achieved.

### Data extraction

Two professional reviewers independently extracted relevant data from texts, tables and figures. Any disagreements in data extraction were resolved through discussion among three authors. The following data was collected: author, published year, country of the study participants, number of patients, age distribution, gender distribution, detection method, cutoff value of ALDH1 expression, rate of high ALDH1 expression, clinicopathological features (T stage, N stage, and tumor differentiation), follow-up time, 5-year OS / DFS rate. Since some publications provided OS/DFS data by Kaplan-Meier curves, but not the rate directly, the software GetData Graph Digitizer 2.25 was used to extract the data.

### Statistical analysis

Software RevMan 5.1 was applied to calculate the OR with the corresponding 95%CI,. The heterogeneity among included studies was evaluated by Q and I^2^ tests, and P-value < 0.1 or I^2^ > 50% indicated the existence of heterogeneity. A random or fixed effects model was applied depending on the heterogeneity analysis. The Egger weighted regression test and Begg rank correlation test were applied to evaluate publication bias. A P value < 0.05 was considered to be statistically significant.

## Results

### Search results

**Fig 1:**
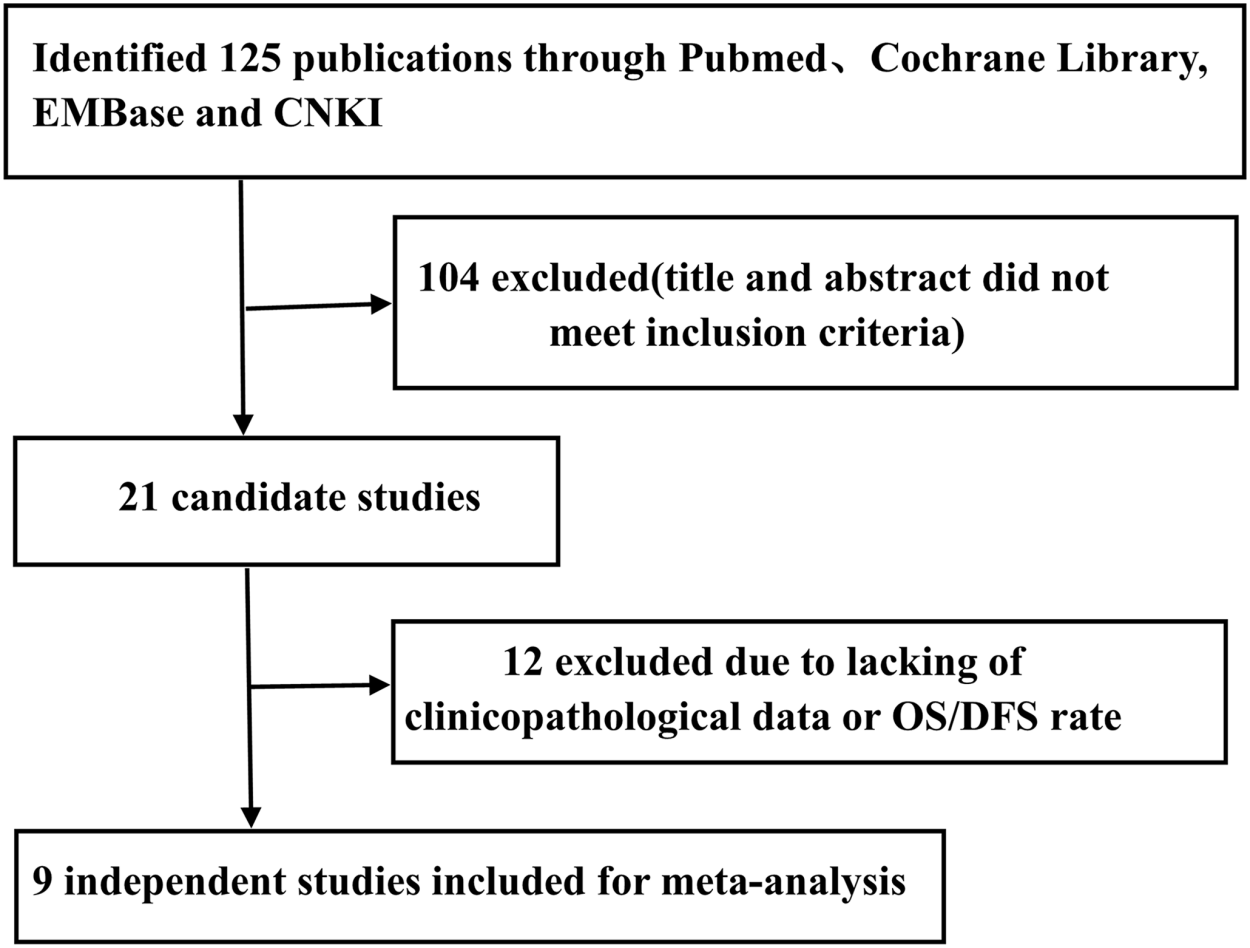
Flowchart of selection of studies for inclusion in this meta-analysis. Nine studies with 1203 patients were included in this meta-analysis. All of these studies investigated the relationship between the expression of ALDH1 and the prognosis of CRC by immunohistochemistry.

Initially, a total of 125 publications were searched according to the search strategy described above. Next, 104 publications were excluded because the studies were not performed in humans, were not original studies (e.g., review, letter), or were not CRC-related studies after browsing the titles and abstracts. Finally, 12 publications were excluded due to the lack of clinicopathological data or OS/DFS rate by reading the full text. Therefore, a total of 9 publications (8 in English and 1 in Chinese) with 1203 patients were included. All of these studies evaluated the correlation between ALDH1 expression and the prognosis of CRC by immunohistochemistry (Fig 1).

### Study characteristics

The main features of the nine included studies are summarized in Table 1. A total of 1203 patients with a median of 98 (range: 21–309) were included. Six publications originated from Asia (four from China, two from Korea), two from American countries (one from the US, one from Brazil), and one from Europe (Netherlands). The 5-year OS was investigated in four studies, and the DFS rate was analyzed in five studies by the Kaplan-Meier method. Five studies reported the correlation between ALDH1 expression and clinicopathological features. The T stage was evaluated in five studies; a median of 35.7% (range: 10.4–54.7%) of patients were stage T1/2, while the other 64.3% (range: 45.3–89.6%) of patients were stage T3/4. The N stage and the grade of tumor differentiation were evaluated in four studies; approximately 53.9% (range: 40.8–64.3%) of patients were identified as lymph node metastasis stage N1/2, while 29.2% (range: 6.3–41.8%) of patients were identified as having poorly differentiated (G3) tumors. In one study [17], 135 patients were treated with radiochemotherapy (RCT) plus surgery, and 74 patients were treated with surgery only. In another study [14], 21 patients with locally advanced rectal adenocarcinoma underwent standardised neoadjuvant RCT and quality-assessed curative TME surgery. Moreover, 51 patients with middle and lower rectal cancer were treated with preoperative RCT [18]. In an additional study, 23 of 231 patients were identified as having mucinous adenocarcinoma, and all the cases received surgery or postoperative RCT [19]. The median percentage (>1.6%) of tumor cells with ALDH1 positive was used as the cutoff value of ALDH1 expression in another study, and 76 of 309 patients received preoperative neoadjuvant chemotherapy [16]. The patients of the remaining four studies were treated with surgery or postoperative RCT, but not preoperative neoadjuvant RCT (Table 1)[20,21,22,23].

**Table 1:** Characteristics of the included studies.

NO.	Author	Year	Country	Case	ALDH1–high (%)	Method	Cutoff	T category T1,2/T3,4	N category N0/N1,2	Differentiation G3/G1,2	Duration of follow-up	5-year OS (%)	5-year DFS (%)
**1**	**Yi Hou**	**2013**	**China**	**98**	**76.5**	**IHC**	**>5%**	**H22/53**	**H22/53**	**H36/39**	**1693**	**H49/75**	**NA**
								**L13/10**	**L13/10**	**L5/18**	**days**	**L18/23**	
**2**	**Avoranta**	**2012**	**Brazil**	**209**	**71.3**	**IHC**	**>3%**	**NA**	**NA**	**NA**	**51.6**	**NA**	**H105/156**
											**months**		**L46/53**
**3**	**Y Deng**	**2013**	**China**	**21**	**71.4**	**IHC**	**>20%**	**NA**	**NA**	**NA**	**36**	**NA**	**H7/15**
											**months**		**L6/6**
**4**	**FEI ZHOU**	**2013**	**China**	**60**	**51.7**	**IHC**	**>20%**	**H7/24**	**H14/17**	**H14/17**	**5**	**NA**	**H7/31**
								**L17/12**	**L12/17**	**L6/23**	**years**		**L19/29**
**5**	**YEOP OH**	**2015**	**Korea**	**51**	**41.2**	**IHC**	**>50%**	**NA**	**NA**	**NA**	**46.5**	**H13/21**	**H17/21**
											**months**	**L27/30**	**L23/30**
**6**	**HUN KIM**	**2013**	**Korea**	**231**	**18.2**	**IHC**	**score>3**	**H4/38**	**H13/29**	**H1/34**	**76**	**NA**	**NA**
								**L20/169**	**L100/89**	**L12/161**	**months**		
**7**	**Shan Deng**	**2010**	**America**	**148**	**31.1**	**IHC**	**>20%**	**NA**	**NA**	**NA**	**101**	**H17/46**	**H22/46**
											**months**	**L48/102**	**L65/102**
**8**	**Beumer**	**2014**	**Nether-lands**	**309**	**49.8**	**IHC**	**>1.6%**	**H81/73**	**NA**	**NA**	**7.7**	**NA**	**NA**
							**median**	**L88/67**			**years**		
**9**	**Zhao-jun**	**2009**	**China**	**76**	**34.2**	**IHC**	**Q score**	**H1/25**	**H15/11**	**H7/19**	**1year-94**	**H8/26**	**NA**
							**>120**	**L10/40**	**L30/20**	**L12/38**	**months**	**L34/50**	

**H: high expression; L: low expression; NA; not available**

### Impact of ALDH1 expression on the 5-year OS and DFS of CRC

**Fig 2:**
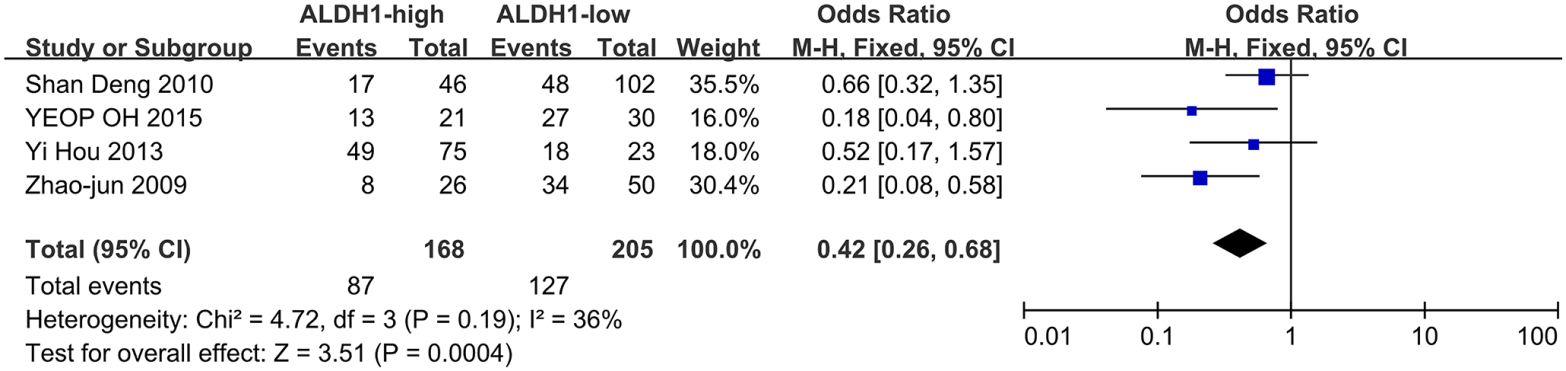
ALDH1 expression and the 5-year OS rate. Four included studies investigated the correlation between ALDH1 expression and OS. High ALDH1 expression was highly correlated with a poor OS (OR = 0.42, 95%CI: 0.26–0.68, P < 0.0004, fixed effects model).

**Fig 3:**
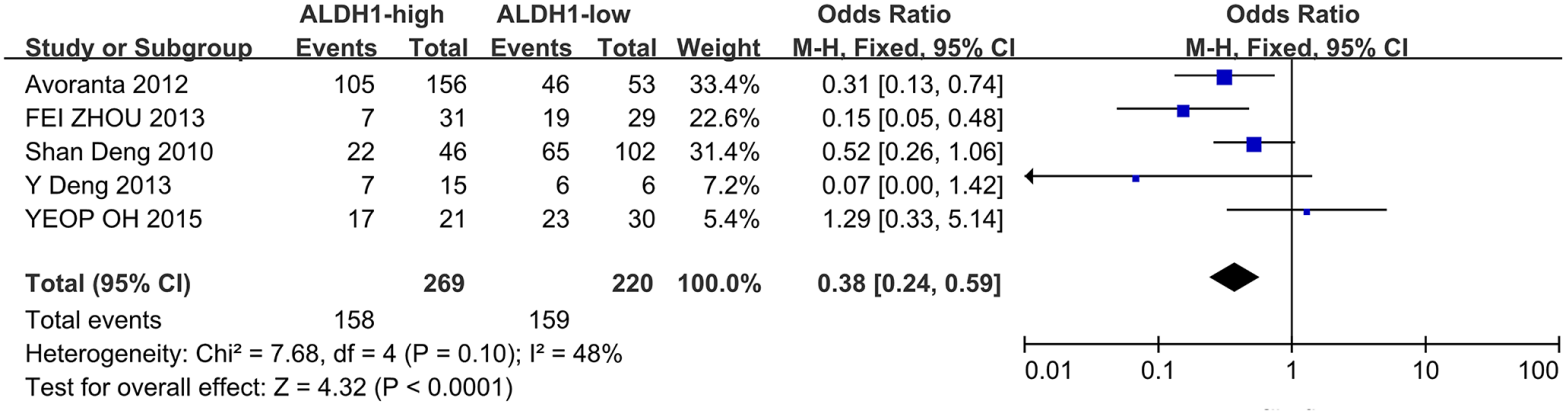
ALDH1 expression and the 5-year DFS rate. Five included studies investigated the correlation between ALDH1 expression and DFS. High ALDH1 expression was highly correlated with a poor DFS (OR = 0.38, 95%CI: 0.24–0.59, P < 0.0001, fixed effects model).

Four of the included studies evaluated the relationship between ALDH1 expression and OS, while five studies analyzed the correlation between ALDH1 expression and DFS. The OR value was measured by Software RevMan 5.1. The heterogeneity analysis was not significant among the eligible studies, and a fixed model was applied to calculate the pooled OR. High ALDH1 expression was significantly correlated with a poor OS and DFS (Fig 2, OR = 0.42, 95%CI: 0.26–0.68, P = 0.0004; Fig 3, OR = 0.38, 95%CI: 0.24–0.59, P < 0.0001, respectively). In fact, all the included studies concluded that high ALDH1 expression is a poor prognostic factor in CRC, except for the study by YEOP Oh *et al*.[18].

### Correlation of ALDH1 expression with clinicopathological features

**Fig 4:**
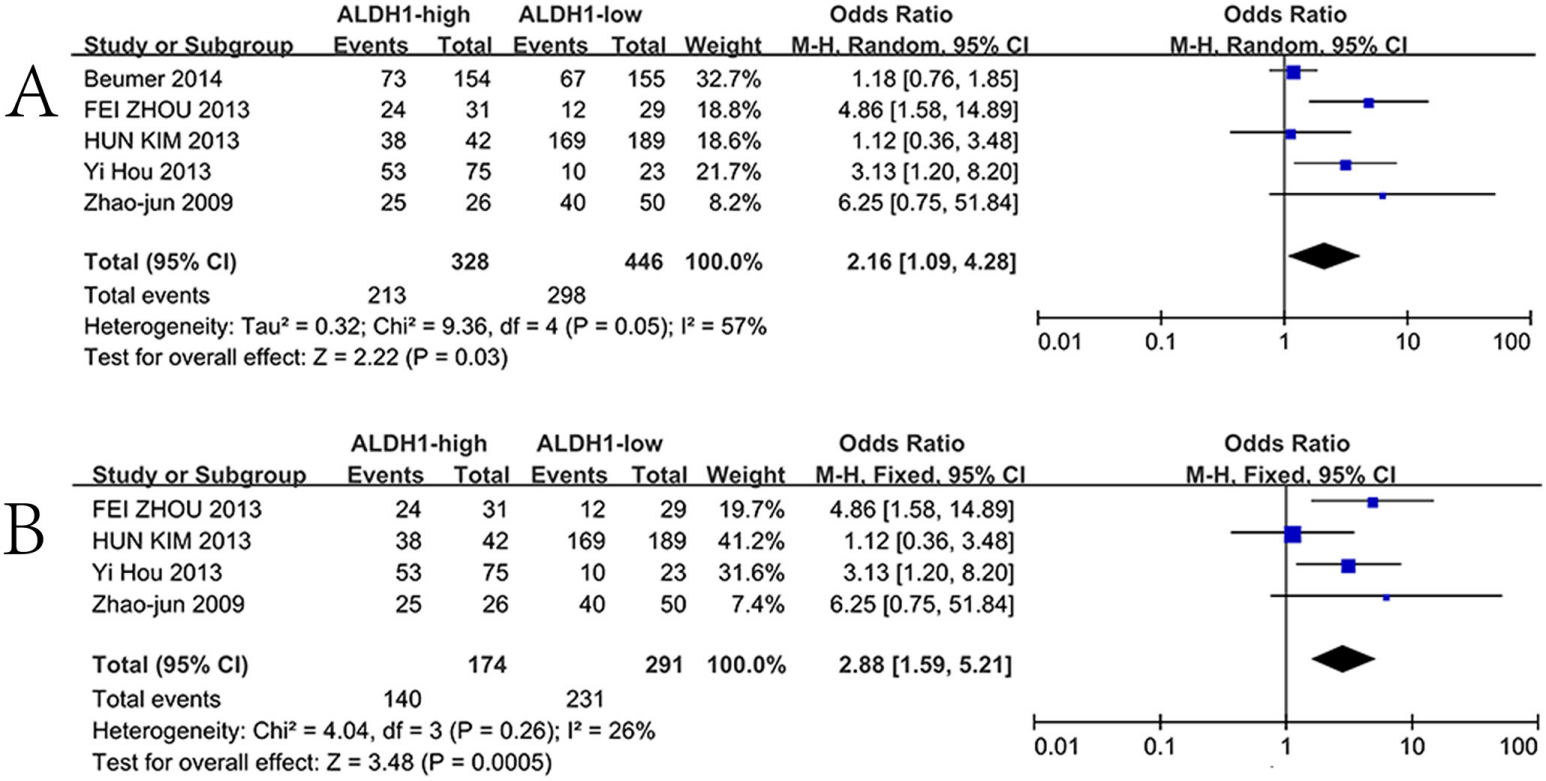
ALDH1 expression and T stage. **A**: Five included studies investigated the correlation between ALDH1 expression and the T stage. High ALDH1 expression was highly correlated with the T stage (T3 + T4 vs. T1 + T2, OR = 2.16, 95%CI: 1.09–4.28, P = 0.03, random effects model). **B**: A subgroup analysis by ethnicity among eastern populations. High ALDH1 expression was highly correlated with the T stage (T3 + T4 vs. T1 + T2, OR = 2.88, 95%CI: 1.59–5.21, P = 0.0005, fixed effects model)

Five included studies investigated the correlation of ALDH1 expression with the T stage. The heterogeneity test was significant among the five studies, and a random effects model was applied to measure the OR. High ALDH1 expression was closely correlated with the T stage (Fig 4A, T3 + T4 vs. T1 + T2; OR = 2.16, 95%CI: 1.09–4.28, P = 0.03). To eliminate the heterogeneity among the above five included studies, a subgroup analysis was performed. One study among western populations was excluded and the other four studies among eastern populations were performed a subgroup analysis. The result showed that the heterogeneity disappeared among the four studies, and a fixed model was applied to calculate the pooled OR. High ALDH1 expression among eastern populations was correlated with the T stage (Fig 4B, T3 + T4 vs. T1 + T2; OR = 2.88, 95%CI: 1.59–5.21, P = 0.0005)

**Fig 5:**
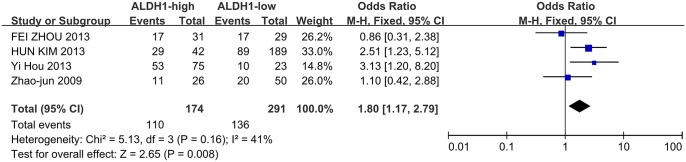
ALDH1 expression and N stage. Four studies investigated the correlation between ALDH1 expression and the N stage. High ALDH1 expression was also associated with a positive N stage (N1/2) (N1 + N2 vs. N0, OR = 1.80, 95%CI: 1.17–2.79, P = 0.008, fixed effects model).

**Fig 6:**
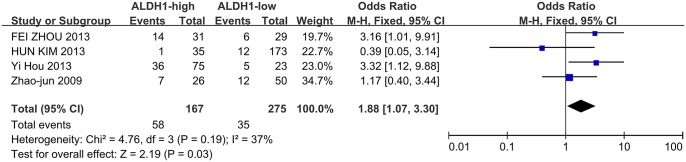
ALDH1 expression and tumor differentiation. Four studies investigated the correlation between ALDH1 expression and the degree of differentiation. High ALDH1 expression was also associated with poor differentiation (G3) (G3 vs. G1 + G2, OR = 1.88, 95%CI: 1.07–3.30, P = 0.03, fixed effects model).

**Fig 7:**
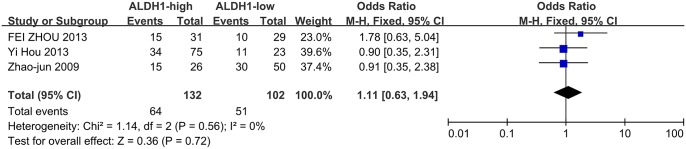
ALDH1 expression and patient age. Three studies investigated the correlation between ALDH1 expression and age. High ALDH1 expression was not associated with age (>60 years old vs. <60 years old, OR = 1.11, 95%CI: 0.63–1.94, P = 0.72, fixed effects model).

Meanwhile, four studies investigated the correlation between ALDH1 expression and the N stage as well as the degree of differentiation. High ALDH1 expression was correlated with a positive N stage (N1/2) and poor differentiation (G3), leading to OR values of 1.8(95%CI: 1.17–2.79, P = 0.008, Fig 5) and 1.88 (95%CI: 1.07–3.30, P = 0.03, Fig 6). However, ALDH1 expression was not correlated with patient age (Fig 7, OR = 1.11, 95%CI: 0.63–1.94, P = 0.72) among the three included studies.

We also performed subgroup analysis by ethnicity(western and eastern populations), tumor location(colon and rectum) among all included studies. Significant association was detected in all stratified analysis (Table 2). The subgroup analysis result showed that high ALDH1 expression in eastern CRC and colon cancer patients was correlated with poor OS (OR = 0.29, 95%CI: 0.15–0.57, P = 0.0003; OR = 0.33, 95%CI: 0.15–0.69, P = 0.003, respectively), T stage (T3+T4) (OR = 2.88, 95%CI: 1.59–5.21, P = 0.0005; OR = 2.39, 95%CI: 1.19–4.80, P = 0.01, respectively), N stage (N1/2) (OR = 1.8, 95%CI: 1.17–2.79, P = 0.008; OR = 2.14, 95%CI: 1.31–3.48, P = 0.002, respectively), which were consistent with the results derived from overall analysis. High ALDH1 expression was also correlated with poor differentiation in eastern CRC patients (OR = 1.88, 95%CI: 1.07–3.30, P = 0.03), but not in colon cancer patients (OR = 1.57, 95%CI: 0.81–3.02, P = 0.18). High ALDH1 expression was correlated with poor DFS in western CRC patients (OR = 0.41, 95%CI: 0.24–0.71, P = 0.001), but not in eastern CRC patients (OR = 0.43, 95%CI: 0.05–3.46, P = 0.43).

**Table 2:** Subgroup analysis of the studies reporting the prognostic value of ALDH1 expression on OS/DFS/T stage/N stage/Differentiation/Age of CRC.

Stratified analysis	Studies	Odds ratio		Model	Heterogeneity	
		OR(95%CI)	P_OR_		I^2^(%)	P
**OS**	4	0.42(0.26–0.48)	0.0004	Fixed	36	0.19
**Ethnicity**						
Western	1	0.66(0.32–1.35)	0.25	Fixed	-	-
Eastern	3	0.29(0.15–0.57)	0.0003	Fixed	0	0.39
**Location**						
Colon	2	0.33(0.15–0.69)	0.003	Fixed	30	0.23
Rectum	1	0.18(0.04–0.80)	0.02	Fixed	-	-
**DFS**	5	0.38(0.24–0.59)	<0.0001	Fixed	48	0.10
**Ethnicity**						
Western	2	0.41(0.24–0.71)	0.001	Fixed	0	0.37
Eastern	2	0.43(0.05–3.46)	0.43	Random	82	0.02
**Location**						
Colon	1	0.52(0.26–1.06)	0.07	Fixed	-	-
Rectum	2	0.57(0.14–2.27)	0.43	Random	66	0.09
**T stage**	5	2.16(1.09–4.28)	0.03	Random	57	0.05
**Ethnicity**						
Western	1	1.18(0.76–1.85)	0.46	Fixed	-	-
Eastern	4	2.88(1.59–5.21)	0.0005	Fixed	26	0.26
**Location**						
Colon	3	2.39(1.19–4.80)	0.01	Fixed	29	0.25
Rectum	0	-	-	-	-	-
**N stage**	4	1.80(1.17–2.79)	0.008	Fixed	41	0.16
**Ethnicity**						
Western	0	-	-	-	-	-
Eastern	4	1.80(1.17–2.79)	0.008	Fixed	41	0.16
**Location**						
Colon	3	2.14(1.31–3.48)	0.002	Fixed	24	0.27
Rectum	0	-	-	-	-	-
**Differentiation**	4	1.88(1.07–3.30)	0.03	Fixed	37	0.19
**Ethnicity**						
Western	0	-	-	-	-	-
Eastern	4	1.88(1.07–3.30)	0.03	Fixed	37	0.19
**Location**						
Colon	3	1.57(0.81–3.02)	0.18	Fixed	48	0.15
Rectum	0	-	-	-	-	-
**Age**	3	1.11(0.63–1.94)	0.72	Fixed	0	56
**Ethnicity**						
Western	0	-	-	-	-	-
Eastern	3	1.11(0.63–1.94)	0.72	Fixed	0	0.56
**Location**						
Colon	2	0.91(0.46–1.77)	0.78	Fixed	0	0.99
Rectum	1	1.78(0.63–5.04)	0.28	Fixed	-	-

P_OR_: P value for odds ratio

### Publication bias

**Fig 8:**
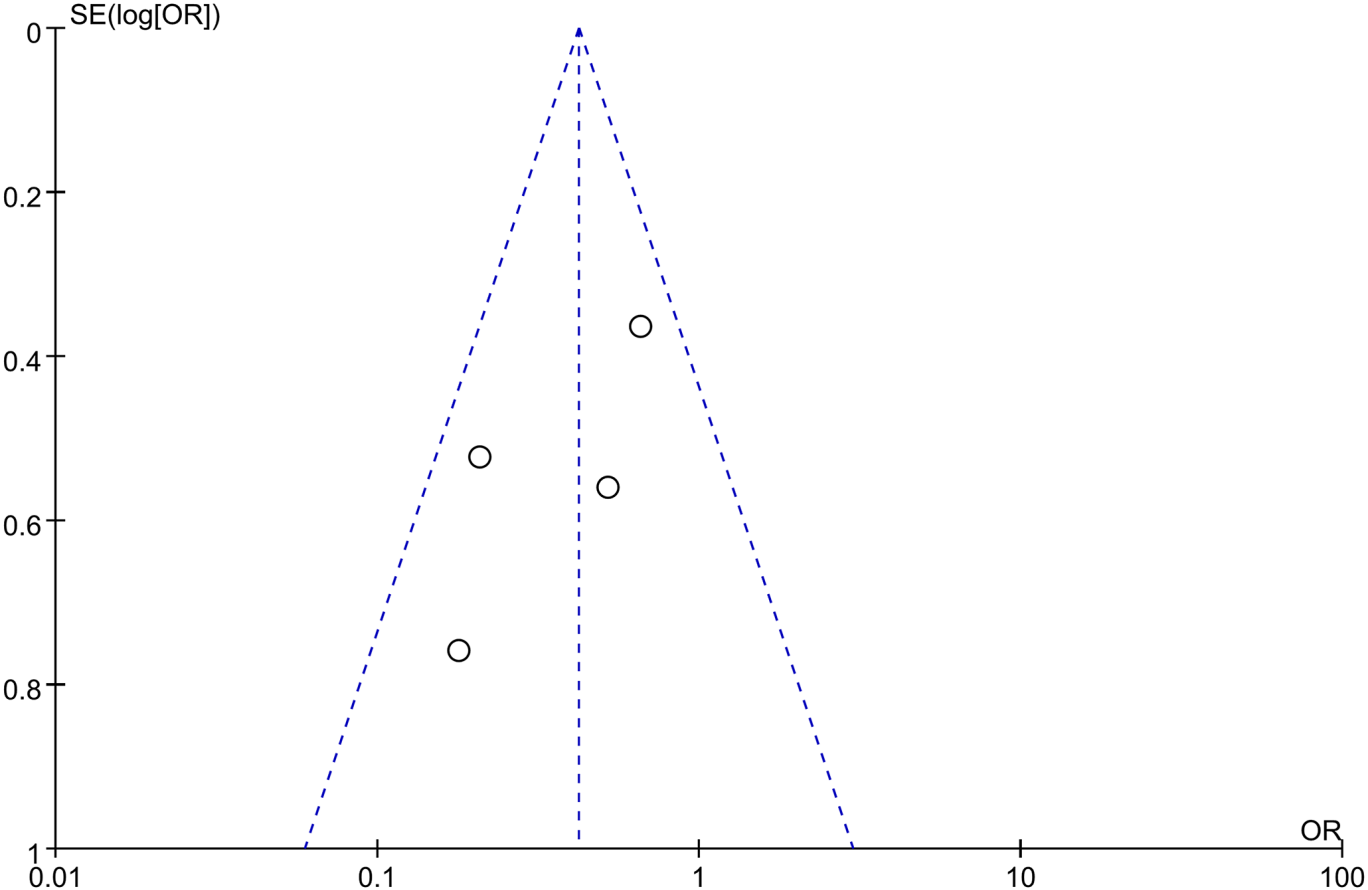
Funnel plot of studies used in the analysis of the 5-year OS rate of CRC. The shape of the funnel plots was almost symmetric, indicating that there was no evidence of publication bias among the publications describing the 5-year OS rate.

**Fig 9:**
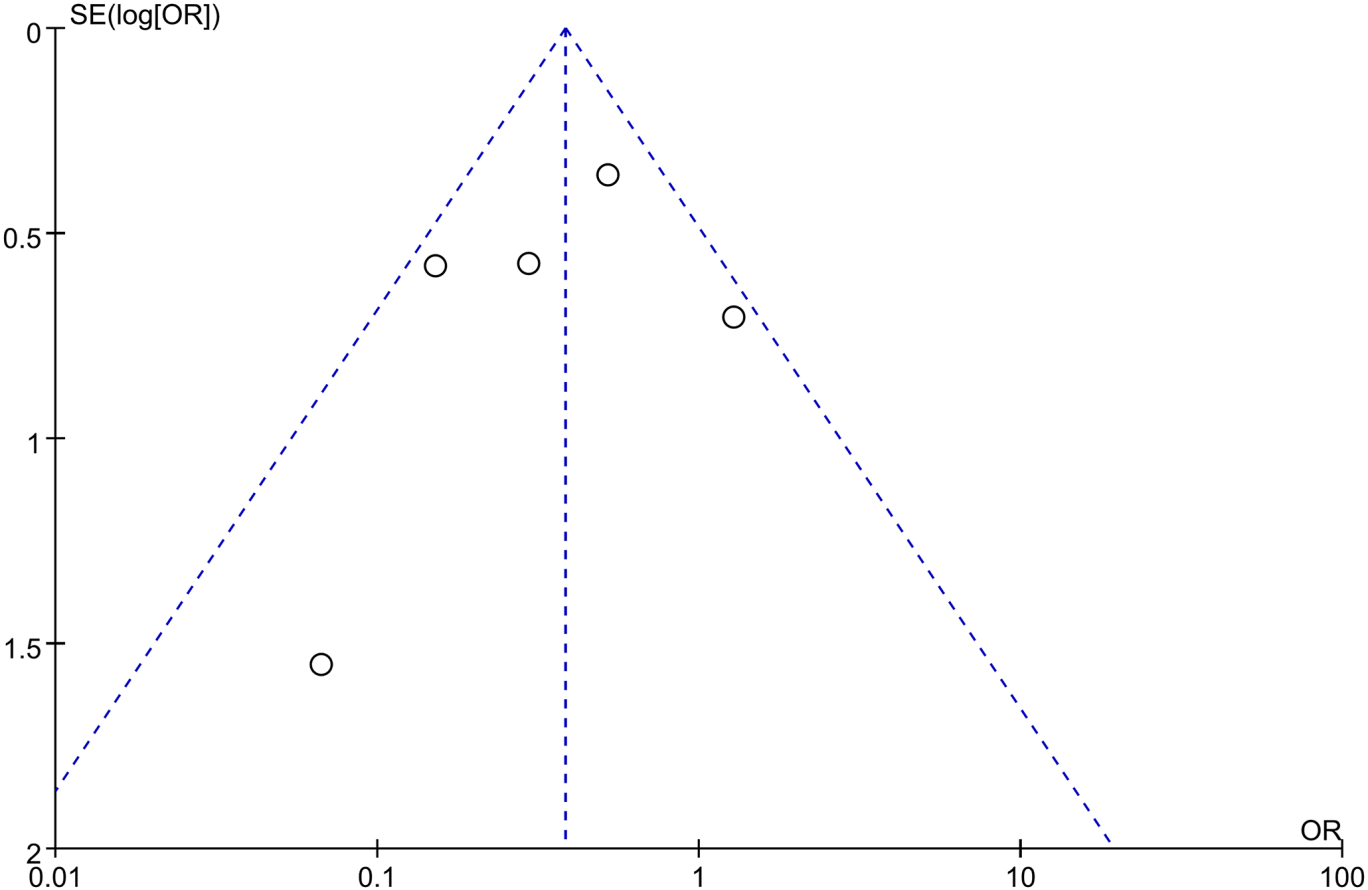
Funnel plot of studies used in the analysis of the 5-year DFS rate of CRC. The shape of the funnel plots was almost symmetric, indicating that there was no evidence of publication bias among the publications describing the 5-year DFS rate.

Publication bias analyses among the included studies were performed by combining the funnel plots of RevMan 5.1 with Begg’s and Egger’s tests using Stata software. The results indicated that the shape of the funnel plots was almost symmetric (Figs 8 and 9). And all the P values > 0.05 in Begg’s and Egger’s tests (Table 3), respectively. Thus, there was no evidence suggesting a publication bias in this study.

**Table 3:** Publication bias analyses among included studies.

Clinicopathological feature	Publication bias	
	*P* values of Begg’s test	*P* values of Egger’s test
T category (T1/2 vs. T3/4)	0.806	0.138
N category (N0 vs. N1/2)	0.734	0.407
Grade (G3 vs. G1/2)	0.734	0.312
Age (<60 years old vs. >60 years old)	0.296	0.159
5-year OS	0.435	0.124
5-year DFS	0.941	0735

P-value < 0.05 is considered statistically significant.

## Discussion

In the past decades, it is believed that tumors are maintained by their own CSCs, which are responsible for cancer metastasis and recurrence [24,25]. Therefore, CSC markers are used to identify CSCs to study their effect in the occurrence and development of tumors [10]. ALDH1 is one of the potential CSC markers that has been identified in recent years. This study is the first to systematically investigate the correlation of ALDH1 expression with the prognosis in CRC. Previous studies mainly focused on the prognostic value of ALDH1 in breast, head and neck carcinoma patients [13][9].

ALDH1 is a type of detoxifying enzyme, which contributes to the oxidation of intracellular aldehydes [26]. The detoxifying capacity of ALDH1 may underlie the recognized longevity of CSCs by protecting themselves against oxidative damage [10]. Many studies have shown that ALDH1 is expressed to different degrees in almost all cancer tissues [7,9,13]. In addition, breast CSCs with high ALDH1 expression show a high tumorigenic capacity and are able to form tumors with 20 cells [27].

Emina *et al*.[10] have shown that ALDH1 activity is applied to isolate CSCs from CRC tissue as well as from other carcinomas, including lung and breast cancer, and found that the proportion of cells that expressed ALDH1 was 3.5 ± 1.0% in CRC. Their study also showed that the ALDH1-positive population is a subset of the CD133-positive population. ALDH1 expression was the highest in peritumoral crypt cells, and ALDH1-positive cells grew more efficiently than ALDH1-negative cells. Our study indicated that the expression of CSC markers such as Lgr5, CD133, and ALDH1 was increased in spherical cells with stem-like properties from DLD-1 cells [28]. Cell proliferation and motility were decreased when knocking down ALDH1 by siRNA. On the contrary, high expression of ALDH1 increased cell proliferation, resistance to chemotherapeutic agents, and tumorigenesis [29].

Although many studies have reported the correlation between ALDH1 expression and the outcomes of CRC patients, the prognostic significance of ALDH1 for CRC remains controversial. In this studies, ALDH1 appeared to have a median expression rate of 46.5% (range: 18.2–76.5%) by immunohistochemistry in CRC patients. Many studies have indicated that ALDH1-high expression is related to poor outcomes, particularly in breast and prostate cancer [30,31,32]. Conversely, it has been shown that low expression of ALDH1 indicates a poor outcomes in pancreatic cancer [33]. Conclusions regarding the relationship between ALDH1 and CRC are also different.

ALDH1 expression of stage III rectal cancer shows a more aggressive feature and can be stratified into different survival groups [18]. It also has been suggested that ALDH1 is a prognostic indicator for rectal cancer patients in stage II–III after treating with RCT [14]. The expression of ALDH1 is higher in stage III–IV colorectal adenocarcinomas [21]. A study involving 98 patients has demonstrated that ALDH1-high expression was significantly associated with the TNM stage, tumor grade, lymph node metastasis, and survival time [20]. In addition, ALDH1 is considered to be a valuable marker to predict the biological behavior and trend of CRC metastasis. However, patients with high ALDH1 expression showed a poor outcome and CD133 expression was correlated with lymph node status, but ALDH1 was not [21]. Meanwhile, ALDH1 expression indicated a poor prognosis and chemotherapy resistance in node-negative rectal cancer patients [17]. Moreover, ALDH1 has been strongly associated with OS, but not with age, race, grade, or number of lymph node metastases [34]. However, another study found that there is no relationship between ALDH1 expression and survival time [35]. Similarly, a study by Lugli showed that the ALDH1-high expression rate was 23.3% and that it was related to the tumor grade but not to differences in survival time [36]. Thus, further studies are needed to draw a definite conclusion.

The current meta-analysis showed that high ALDH1 expression as an independent prognostic factor was significantly associated with the OS and DFS rates, T stage, N stage, and tumor differentiation, but not with the patient age. These results are consistent with previous studies [20,21]. ALDH1 as a CSC marker increases the capacity of cell proliferation and invasion; therefore, ALDH1-positive patients are associated with greater depth of invasion, lymph node metastasis, and poor differentiation, which may reflect the biological behavior of tumors.

Meanwhile, we found that the expression of ALDH1 is unstable and varies among different populations. But it is unclear how stable the expression of CSC markers is and how they are influenced by some complicated mechanism. It is widely believed that the differences of ALDH1 expression and prognosis exists in CRC between western and eastern populations [13]. In our study, there were 537 patients from eastern countries and 666 patients from western countries. In addition, the expression rate of ALDH1 among the western populations was higher than that among the eastern populations (P < 0.00001), which were 52.4% (349/666) and 39.1% (210/537), respectively. Our study showed that high ALDH1 expression was correlated with poor OS in eastern CRC patients and with poor DFS in western CRC patients. This may be a potential factor leading to different outcomes among western and eastern CRC populations.

The expression of ALDH1 may be up-regulated after neoadjuvant RCT. For instance, YEOP Oh *et al*.[18] have reported that high expression for ALDH1 was 20.4% before neoadjuvant therapy and 41.2% after neoadjuvant RCT. They explained that this difference may be related to the drug resistance activity of ALDH1-positive cells. ALDH1 also may be up-regulated due to inflammation or hypoxia. Another study has demonstrated that ALDH1-high expression is increased in inflammatory bowel disease(IBD), indicating an important role for CSCs in the progression to CRC in patients with IBD [37].

Moreover, ALDH1 as a CSC marker is commonly co-expressed with other CSC markers (e.g., CD24, CD44, and CD133) as well as the anti-apoptotic molecules Bcl-2 and ABCG2 [38–39]. In addition, the combination of these markers may provide a better selection of CSCs [40]. For example, combining ALDH1, EpCAM, and survivin as strong prognostic factors for survival has been investigated in CRC [16]. Thus, the proposed bio-marker combination should be further studied for applying in clinical settings.

However, there are some limitations in this study. First, the number of included studies and case samples in each study are relative small, which may reduce the power and accuracy of subgroup analysis. Second, the cutoff values of high ALDH1 expression with immunohistochemistry are different among the included studies, ranging from >1.3% to >50% of positively stained cells. In addition, the differences of cutoff values may contribute to the observed heterogeneity. Thus, standardizing the cutoff value and unifying the detection method to classify the level of ALDH1 expression as “high” or “low” are needed. Lastly, the CRC patients were treated and investigated at different times and received different treatments.

The potential publication bias is inevitable. We need to consider the fact that studies with positive results are easily accepted, whereas studies with negative results are often rejected. Although we had tried to collect all relevant data, some data were still missing. The studies included in our meta-analysis were restricted to only articles published in English or Chinese, and the number was relatively small. Using single method to detect the publication bias will be limited. Therefore we combined various methods (funnel plots, Begg and Egger test) to detect the publication bias to increase the reliability of the result. However, the relevant limitation in this study may still reduce the power and accuracy of subgroup analysis. It may exaggerate treatment effect or intensity of associated risk factors. The above limitations may partially influence the significance of ALDH1 expression in the survival and the clinicopathological analyses. Therefore, larger prospective studies are needed to validate our results.

In summary, this meta-analysis indicates that ALDH1 as a CSC marker is an important predictor of a poor outcome and CRC progression. Our results suggest that high ALDH1 expression is correlated with clinicopathological features of CRC, such as the T stage, N stage, and tumor differentiation, but not with the patient age. Moreover, ALDH1 is an independent factor associated with decreased survival. Thus, further studies of ALDH1 and its potential role as a CSC marker for CRC prognosis are needed.

## Supporting Information

S1 PRISMA Checklist PRISMA Checklist. (DOC)

S1 Table Characteristics of the included studies. (DOC)

S2 Table Subgroup analysis of the studies reporting the prognostic value of ALDH1 expression on OS/DFS/T stage/N stage/Differentiation/Age of CRC. (DOC)

S3 Table Publication bias analyses among included studies. (DOC)
