# Supplementary material for: Prognostic Value of Cancer Stem Cell Marker ALDH1 Expression in Colorectal Cancer: A Systematic Review and Meta-Analysis
Source: PLoS One. 2015 Dec 18;10(12):e0145164. doi: 10.1371/journal.pone.0145164 (PMC4686173; doi:10.1371/journal.pone.0145164)
Supplement: S1 Table — (DOC) [file pone.0145164.s002.doc]

| **Table 1. Characteristics of the included studies** | | | | | | | | | | | | | |
| --- | --- | --- | --- | --- | --- | --- | --- | --- | --- | --- | --- | --- | --- |
| **NO.** | **Author** | **Year** | **Country** | **Case** | **ALDH1**  **-high**  **(%)** | **Method** | **Cutoff** | **T category**  **T1,2/T3,4** | **N**  **category**  **N0/N1,2** | **Differentiation**  **G3/G1,2** | **Duration of follow**  **-up** | **5-year OS**  **(%)** | **5-year**  **DFS**  **(%)** |
| **1**  **2**  **3**  **4**  **5**  **6**  **7**  **8**  **9** | **Yi Hou**  **Avoranta**  **Y Deng**  **FEI ZHOU**  **YEOP OH**  **HUN KIM**  **Shan Deng**  **Beumer**  **Zhao-jun** | **2013**  **2012**  **2013**  **2013**  **2015**  **2013**  **2010**  **2014**  **2009** | **China**  **Brazil**  **China**  **China**  **Korea**  **Korea**  **America**  **Nether-**  **lands**  **China** | **98**  **209**  **21**  **60**  **51**  **231**  **148**  **309**  **76** | **76.5**  **71.3**  **71.4**  **51.7**  **41.2**  **18.2**  **31.1**  **49.8**  **34.2** | **IHC**  **IHC**  **IHC**  **IHC**  **IHC**  **IHC**  **IHC**  **IHC**  **IHC** | **>5%**  **>3%**  **>20%**  **>20%**  **>50%**  **score>3**  **>20%**  **>1.6%**  **median**  **Q score**  **>120** | **H22/53**  **L13/10**  **NA**  **NA**  **H7/24**  **L17/12**  **NA**  **H4/38**  **L20/169**  **NA**  **H81/73**  **L88/67**  **H1/25**  **L10/40** | **H22/53**  **L13/10**  **NA**  **NA**  **H14/17**  **L12/17**  **NA**  **H13/29**  **L100/89**  **NA**  **NA**  **H15/11**  **L30/20** | **H36/39**  **L5/18**  **NA**  **NA**  **H14/17**  **L6/23**  **NA**  **H1/34**  **L12/161**  **NA**  **NA**  **H7/19**  **L12/38** | **1693**  **days**  **51.6**  **months**  **36**  **Months**  **5**  **years**  **46.5**  **months**  **76**  **months**  **101**  **months**  **7.7**  **Year**  **1year-94**  **months** | **H49/75**  **L18/23**  **NA**  **NA**  **NA**  **H13/21**  **L27/30**  **NA**  **H17/46**  **L48/102**  **NA**  **H8/26**  **L34/50** | **NA**  **H105/156**  **L46/53**  **H7/15**  **L6/6**  **H7/31**  **L19/29**  **H17/21**  **L23/30**  **NA**  **H22/46**  **L65/102**  **NA**  **NA** |
| **H: high expression; L: low expression; NA; not available** | | | | | | | | | | | | | |
