# Supplementary material for: Prognostic Value of Cancer Stem Cell Marker ALDH1 Expression in Colorectal Cancer: A Systematic Review and Meta-Analysis
Source: PLoS One. 2015 Dec 18;10(12):e0145164. doi: 10.1371/journal.pone.0145164 (PMC4686173; doi:10.1371/journal.pone.0145164)
Supplement: S2 Table — (DOC) [file pone.0145164.s003.doc]

**Table 2 Subgroup analysis of the studies reporting the prognostic value of ALDH1 expression on OS/DFS/T stage/N stage/Differentiation/Age of CRC**

| Stratified | Studies | | Odds ratio |  | Model | Heterogeneity | |
| --- | --- | --- | --- | --- | --- | --- | --- |
| analysis | |  | OR(95%CI) | P_OR_ |  | I^2^(%) | P |
| **OS** | | 4 | 0.42(0.26-0.48) | 0.0004 | Fixed | 36 | 0.19 |
| **Ethnicity** | |  |  |  |  |  |  |
| western | | 1 | 0.66(0.32-1.35) | 0.25 | Fixed | - | - |
| Eastern  **Location**  Colon  Rectum  **DFS**  **Ethnicity**  western  Eastern  **Location**  Colon  Rectum  **T stage**  **Ethnicity**  western  Eastern  **Location**  Colon  Rectum  **N stage**  **Ethnicity**  western  Eastern  **Location**  Colon  Rectum  **Differentiation**  **Ethnicity**  western  Eastern  **Location**  Colon  Rectum  **Age**  **Ethnicity**  western  Eastern  **Location**  Colon  Rectum | | 3  2  1  5  2  2  1  2  5  1  4  3  0  4  0  4  3  0  4  0  4  3  0  3  0  3  2  1 | 0.29(0.15-0.57)  0.33(0.15-0.69)  0.18(0.04-0.80)  0.38(0.24-0.59)  0.41(0.24-0.71)  0.43(0.05-3.46)  0.52(0.26-1.06)  0.57(0.14-2.27)  2.16(1.09-4.28)  1.18(0.76-1.85)  2.88(1.59-5.21)  2.39(1.19-4.80)  -  1.80(1.17-2.79)  -  1.80(1.17-2.79)  2.14(1.31-3.48)  -  1.88(1.07-3.30)  -  1.88(1.07-3.30)  1.57(0.81-3.02)  -  1.11(0.63-1.94)  -  1.11(0.63-1.94)  0.91(0.46-1.77)  1.78(0.63-5.04) | 0.0003  0.003  0.02  <0.0001  0.001  0.43  0.07  0.43  0.03  0.46  0.0005  0.01  -  0.008  -  0.008  0.002  -  0.03  -  0.03  0.18  -  0.72  -  0.72  0.78  0.28 | Fixed  Fixed  Fixed  Fixed  Fixed  Random  Fixed  Random  Random  Fixed  Fixed  Fixed  -  Fixed  -  Fixed  Fixed  -  Fixed  -  Fixed  Fixed  -  Fixed  -  Fixed  Fixed  Fixed | 0  30  -  48  0  82  -  66  57  -  26  29  -  41  -  41  24  -  37  -  37  48  -  0  -  0  0  - | 0.39  0.23  -  0.10  0.37  0.02  -  0.09  0.05  -  0.26  0.25  -  0.16  -  0.16  0.27  -  0.19  -  0.19  0.15  -  0.56  -  0.56  0.99  - |

P_OR_: P value for odds ratio
