# Supplementary material for: Prognostic Value of Cancer Stem Cell Marker ALDH1 Expression in Colorectal Cancer: A Systematic Review and Meta-Analysis
Source: PLoS One. 2015 Dec 18;10(12):e0145164. doi: 10.1371/journal.pone.0145164 (PMC4686173; doi:10.1371/journal.pone.0145164)
Supplement: S3 Table — (DOC) [file pone.0145164.s004.doc]

| **Table 3. Publication bias analyses among included studies.** | | |
| --- | --- | --- |
| Clinicopathological feature | Publication bias | |
|  | *P* values of Begg’s test | *P* values of Egger’s test |
| T category (T1/2 vs. T3/4) | 0.806 | 0.138 |
| N category (N0 vs. N1/2) | 0.734 | 0.407 |
| Grade (G3 vs. G1/2) | 0.734 | 0.312 |
| Age (<60 years old vs. >60 years old) | 0.296 | 0.159 |
| 5-year OS | 0.435 | 0.124 |
| 5-year DFS | 0.941 | 0735 |
| P-value < 0.05 is considered statistically significant. | | |
